# 

**Published:** 1862-10

**Authors:** 


### DENTAL REGISTER

### OF THE WEST.

EDITED BY

J. TAFT AND GEO. WATT.

OCTOBER,–1862

**MONTHLY:**

**Three Dollars per Annum, in advance.**

CINCINNATI:
J. TAFT, PUBLISHER.

J. TAFT, DENTIST, No. 56 WEST FOURTH STREET.

